# Inhaled nitrous oxide can reduce the pain perception in post Caldwell-Luc operation patients–a randomised trial

**DOI:** 10.1038/s41598-017-15731-9

**Published:** 2017-12-19

**Authors:** Ting Dong, Mingwen Liu, Kun Lv

**Affiliations:** 1 The State Key Laboratory Breeding Base of Basic Science of Stomatology (Hubei-MOST) and Key Laboratory of Oral Biomedicine Ministry of Education, Wuhan, China; 2 Department of Oral and Maxillofacial Trauma and Plastic Aesthetic Surgery, School and Hospital of Stomatology, Wuhan University, Wuhan, 430079 People’s Republic of China

## Abstract

To assess the efficiency of inhaled nitrous oxide (N_2_O) for the reduce the perception pain in extraction of iodoform gauze packing strips in post Caldwell-Luc operation patients. This randomized single blind trial included a total of 47 patients, aged between 14–82 years, in which undergoing radical maxillary sinusotomy. Based on the randomization procedure, patients were treated with (experimental) or without (control) inhaled N_2_O. The visual analogue scale scores (VAS scores) of the pain perception and the headache duration time were comparatively studied. The average VAS score for pain perception was 3.92 in the N_2_O group and 7.87 in the control group. The average headache duration time was 0.12 hour in the N_2_O group and 13.09 hours in the control group. Both VAS scores for pain perception during the treatment and the average headache duration time were significantly reduced in the inhaled N_2_O group (P < 0.01). Reduced pain perception and headache duration time indicated that, the inhaled N_2_O method might be viable in extraction of iodoform gauze packing strips in post Caldwell-Luc operation patients.

## Introduction

The Caldwell-Luc operation is used today as a normal operation for chronic maxillary sinusitis, neoplasms of maxillary sinus and the others^1^. It is still the surgical approach of choice for the odontogenic sinusitis or pathology of odontogenic sinus^2^. Iodoform gauze was used for packing the sinus cavity to prevent immediate postoperative bleeding and facial edema, which was removed on the fifth day after surgery^2^.

Due to the abundant nerve endings of nasal mucosa and maxillary sinus, it could generate a great pain when doctors pull those filling materials out^3^. Usually, the patients feel an extreme pain with a constant headache or even fall into shock, which causing enormous psychophobia fear.

Nitrous oxide (N_2_O) is a colorless and odorless gas with a sweet taste. It is considered as a safe well-tolerated sedative agent with the significant analgesic effect^4^. Rapid onset of action, ease of administration, and favorable cardiorespiratory profile suggest that, N_2_O may be an ideal analgesic agent for extraction of the stuff after the radical maxillary sinusotomy.

The current study was designed to evaluate the analgesic effect of N_2_O in the extraction of iodoform gauze packing strips.

## Materials and Methods

### Patients

The single blinded randomized controlled trial was permitted by the School and Hospital of Stomatology, Wuhan University and the Helsinki Declaration was followed in this study. Informed written consents from of all 47 patients involved in this study were obtained, which was approved by the ethics committee of the School and Hospital of Stomatology, Wuhan University(Clinical trial registration number ChiCTR-ONC-17010890, 2017/3/16, 1008002 Retrospective registration. Inhaled nitrous oxide can reduce the pain perception in extraction of iodoform gauze packing strips in post Caldwell-Luc operation patients). A random numbers table was used to generate the random allocation sequence. Sequentially numbered, opaque, sealed envelopes were used to ensure concealment. Patients undergoing radical maxillary sinusotomy were selected for their eligibility. A nurse generated the random allocation sequence. A senior doctor enrolled and assigned participants to interventions. Patients aged between 14–82 years; male and female; undergoing radical maxillary sinusotomy; present a diagnosis includes odontogenic cyst, maxillary sinusitis, odontogenic tumor was the selected inclusion criteria. Those patients unable to participate in consent procedures or has a coronary artery disease, uncontrolled hypertension and uncontrolled diabetes, pneumothorax, pneumocephalus, chronic obstructive pulmonary disease (COPD) and Bleomycin therapy and Dihydropteridine Reductase (DHPR) Deficiency, vegetarians or any patient that can’t wear the nasal mask for physical or psychological reasons were excluded. Not accompanied by another resection surgery. Otherwise unable to participate in clinical assessments due to a medical condition or medication status. Cases were collected in the Department of Oral and Maxillofacial Surgery of School and Hospital of Stomatology, Wuhan University from March 2013 to August 2017 (Table 1). No changes to methods after trial commencement.

**Table 1:** Subjects Demographics and Group Assignment.

Demographic variables	Control group (n = 23)	N_2_O group (n = 24)
Male	14(60.9%)	14(58.3%)
Female	9(39.1%)	10(41.7%)
Mean age in years (standard deviation; range)	45.35(SD18.66;15–80)	44.46(SD16.36;16–68)
**Marital Status**		
Married	19	18
Single	4	6
**Smoking status**		
*Smoking	5	5
No smoking	18	19
**Present disease**		
Odontogenic cyst	16	15
Maxillary sinusitis	5	6
Odontogenic tumor	2	3
**Drug abuse**	0	0
*Alcohol consumption	3	4
**Prior disease**	19	22
Hypertension	4	4
Diabetes	2	2
Gastric ulcer	2	3
Lim fractures	2	3
Odontogenic cyst	2	2
Others	7	8

*Smoking status or alcohol consumption status more than 5 years.

### Study design

The patients were randomized to two groups with 23 patients in control group and 24 patients in experimental group. Neither of the two groups was informed about the method of manipulation with or without N_2_O. In the control group, no N_2_O was used in extraction of iodoform gauze packing strips except 5 liters per minute flow of oxygen. After pulling out the iodoform gauze packing strips, the control group patients continually inhale the oxygen for another 2 minutes. In the experimental group, the patient inhaled N_2_O for about 15 minutes until the patient feel drowsy^5^. After pulling out the iodoform gauze packing strips, the patient continually inhales the N_2_O for another 2 minutes. In experiment group, the concentration of oxygen was 60% concomitant with 2 liters per minute (LPM) flow of N_2_O for patients. The machine for the N_2_O inhaled anesthesia used in this study was manufactured by Parker Hannifin Corporation Porter Instrument Division in USA.

### Outcomes

The patient’s pain sensation and duration time of headache represented the outcomes of this study. We used the visual analogue scale scores (VAS scores) to record the pain perception during the operation^6^. The VAS scores were set as below: 0 indicate “No Pain”; 10 indicate “Severe Pain”. Constant headache duration time was also recorded for analysis.

### Statistical analysis

Means and standard deviations for VAS scores and headache duration time were calculated and analyzed using SPSS 17.0 software. To test the differences between groups, an independent t-test was used.

## Results

All 47 patients were successfully managed. No complication occurred. A significant statistical difference was presented in the VAS score during the operation between two groups (P < 0.01, Table 2). The average VAS score was 3.92 in the N_2_O group and 7.87 in the control group. A significant statistical difference in headache duration time was also presented between two groups(P < 0.01, Table 2). The average headache duration time was 0.12 hour in the N_2_O group and 13.09 hours in the control group.

**Table 2:** VAS scores of the pain perception during the operation and the average headache duration time after the operation.

Group	VAS scores	Headache duration(h)
Control group (n = 23)	7.87 (0.69)	13.09 (0.85)
N_2_O group (n = 24)	*3.92 (0.72)	*0.12 (0.09)

Note: The outcomes presented in mean and (standard deviation) were analyzed with an independent t test between groups. *P < 0.01.

## Discussions

### The severe pain in extracting the iodoform gauze

The limitations of this trial are the small sample number of participation. The number of enrollment could be increased to improve the precision. In this study, patients in the control group showed great pain (mean 7.87) during the operation, and most of them reported constant headache after the operation. The pathogenesis of headache is very complex and yet hasn’t been explained clearly. It is generated mainly that when the pain receptors inside the intracranial and extracranial hyperalgesia structures get stimulated, and the stimulation translate to cerebral cortex through the pain pathways. Sinus and nasal mucosa are both hyperalgesia structures. Mechanical stimulations on the hyperalgesia structures can cause headache, especially when a strong stimulation exist such as the extraction of iodoform gauze.

### Pain reduced by inhaled nitrous oxide

N_2_O is widely used in dentistry^5,7,8^. N_2_O could relieve patients’ psychological fear and improve their satisfaction. It has a rapid onset because the uptake and distribution of N_2_O to the brain tissue is largely dependent on its low partition coefficients^9,10^. According to the small sample size of this study, it reduced the pain greatly for the extraction of sinus stuffing after the radical maxillary sinusotomy. We presume it has the similar analgesic mechanism.

The mechanism of the analgesic effects is that N_2_O can induces endogenous opioid peptide release by binding to opioid receptors located in the periaqueductal gray matter and noradrenergic neurons of the brainstem. This results in the release of opioid in the brainstem, which in turn inhibits the GABA neurons thereby removing the inhibition on the descending noradrenergic inhibitory pathways. This dis-inhibition of the noradrenergic neurons in the brainstem modulates nociception by releasing norepinephrine into the spinal cord to inhibit pain signaling^11–13^.

### Safety

The efficacy and safety of N_2_O is well established, particularly in the pediatric emergency department. Using N_2_O alone can help facilitate performing painful and/or anxiety-provoking procedures such as the reduction of fractures and repair of lacerations^14^. Nitrous oxide seems safe for children of all ages. It can be safely administered at up to 70% concentration by nasal mask for pediatric procedural sedation, particularly for short procedures^15^. In addition, in very elderly subjects, N_2_O also shows the favorable tolerability^16^.

### The indications and contraindications of nitrous oxide

With its long history of safety in medicine and dentistry, N_2_O sedation can be used safely for almost all patients routinely treated. Some of the most commonly reported negative side effects of N_2_O include voting, nausea, dizziness, headache, tingling and euphoria^17^. It might has side-effects on the immune system, hematologic complications such as megaloblastic anemia, myocardial risk and neurological effects^13^. N_2_O is known to have an abuse potential, although debate regarding the toxic effects of such abuse continues, including myeloneuropathy, subacute combined degeneration, psychosis, low or low-normal Vitamin B12^18^. The contraindications of it include any patient that can’t wear the nasal mask for physical or psychological reasons and any “trapped air” in the body, such as pneumothorax, pneumocephalus, chronic obstructive pulmonary disease (COPD) and other diseases. Bleomycintherapyand Dihydropteridine Reductase (DHPR) Deficiency are also its contraindications^19,20^. Loss of vision was reported caused by expansion of intraocular perfluoropropane (C(3)F(8)) gas during nitrous oxide anesthesia^21^. Overall, The N_2_O is comparatively safe to us, but the abuse can also cause toxicity. Therefore, using of N_2_O should under the correct administration.

### Consequences

Nitrous oxide may inactivate the vitamin B12 with detrimental consequences for folate and methionine metabolism. It could be detectable by an increase in total plasma homocysteine^22^. Thus vitamin B12 levels should be checked in people with risk factors prior to inhalation of N_2_O. Especially vegans, should be screened for the deficiency of vitamin B12 before using N_2_O. Vegetarians should give strong consideration to the use of vitamin B12 supplements to ensure adequate vitamin B12 intake^23^. Symptoms can be treated with high doses of vitamin B12. In addition, protection of the clinicians and nurses is necessary since reduced fertility was found among female dental assistants exposed to high levels of nitrous oxide^24^. Our protocol using only inhaled nitrous oxide is safe.

### Limitation

The small sample size is a limitation in this study. More sound support of the sample size should be provided before widely applied.

## Conclusions

As a safe well-tolerated sedative agent and analgesic, the N_2_O can be used to assist the postoperative treatment of the radical maxillary sinusotomy. By reducing pain perception and headache duration time indicate that, the inhaled N_2_O method might be viable in extraction of iodoform gauze packing strips in post Caldwell-Luc operation patients.
